# Identification of a genetic variant associated with treatment outcome in ovarian cancer: the potential role of cholesterol metabolism as a determinant of response to chemotherapy

**DOI:** 10.1186/1897-4287-10-S2-A36

**Published:** 2012-04-12

**Authors:** Georgia Chenevix-Trench, Yi Lu, Sharon Johnatty, Eric Gamazon, Jonathan Beesley, Xiaoqing Chen, Bo Gao, Paul Harnett, R Stephanie Huang, Evelyn Despierre, Florian Heitz, Estrid Hogdall, Claus Hogdall, Robert Brown, Kirsten Moyisch, Peter Fasching, Ellen Goode, Amanda Russell, Michelle Henderson, Michelle Haber, Eileen Dolan, Stuart Macgregor, Anna deFazio

**Affiliations:** 1 Queensland Institute of Medical Research, Brisbane, Australia; 2 Department of Medicine, University of Chicago, Chicago, IL, USA; 3 Department of Gynaecological Oncology and Westmead Institute for Cancer Research, University of Sydney at the Westmead Millennium Institute, Westmead Hospital, Sydney, Australia; 4 Department of Gynaecologic Oncology, University Hospitals Leuven, Leuven, Belgium; 5 Abteilung für Gynäkologie und gynäkologische Onkologie, Ludwig-Erhardt Str. 100, 65197 Wiesbaden, Germany; 6 Dept. of Virus, Hormones and Cancer, Institute of Cancer Epidemiology, Danish Cancer Society, Copenhagen, Denmark; 7 Imperial College London, Hammersmith Hospital Campus, London, UK; 8 Roswell Park Cancer Center, Buffalo, NY, USA; 9 Division of Hematology and Oncology, Department of Medicine, David Geffen School of Medicine, Los Angeles, California, USA; 10 Department of Health Sciences Research, Mayo Clinic College of Medicine, Rochester, MN, USA; 11 The Children’s Cancer Institute of Australia, Sydney, Australia

## 

Cell-based models have shown that response to chemotherapy has a heritable component. We hypothesized that in order to identify loci associated with treatment outcome we should focus on cases known to have had uniform chemotherapy for epithelial ovarian cancer. We therefore performed a two-stage genome-wide association study (GWAS) of progression free survival (PFS) following first line carboplatin/paclitaxel chemotherapy in ovarian cancer cases. In the first stage, we genotyped (Illumina Omni1) 183 Australian cases selected using an extreme phenotype design, and also included data on 134 cases from the TCGA and 68 from Mayo Clinic. In this stage, 260 cases had ‘uniform’ treatment (“primary” group: at least 4 cycles of carboplatin 5-6 AUC and paclitaxel 135-175 mg/m^2^ every three weeks) and 125 cases (“secondary”) had an unknown amount of carboplatin/paclitaxel chemotherapy. In the 2nd stage we genotyped 156 of the top ranking genotyped and imputed SNPs in 4660 cases (1080 ‘primary’ and 1433 ‘secondary’) from 11 sites in OCAC. The additive allelic association with the PFS was assessed in a Cox Proportional Hazards model, adjusting for study site, histological subtype, grade, stage and residual disease. For the SNPs with low minor allele frequencies, we ran permutations to correct the asymptotic p-values. One SNP clearly replicated in the 2nd stage. The associations in both stages were strongest in the “primary” group (1^st^ stage results for this imputed SNP: hazard Ratio (HR) per minor allele=6.30, 95% CI=[4.26, 9.30], two-sided asymptotic p-value=1.88e-6, permuted p-value=4.46e-5; 2^nd^ stage: HR per minor allele=3.37, 95% CI=[2.48, 4.62], one-sided asymptotic p-value=5e-5, permuted p-value=2.9e-4; meta-analysis p-value corrected by permutation=1.7e-7).

Furthermore, an independent cell-based GWAS conducted using HapMap lymphoblastoid cell lines showed that a more common SNP (MAF~4.5%) in moderate LD with our reported SNP (r^2^~0.28), was associated with carboplatin sensitivity (p-value=9e-3). Both these SNPs are located in a gene on chromosome 9 that is known to be associated with circulating levels of high-density lipoprotein cholesterol. Low levels of expression of this gene are associated with poor outcome for both progression free (P=.017) and overall survival (P=.001) from serous ovarian cancer in The Cancer Genome Atlas.

We are now genotyping a panel of rare and common SNPs in this gene in additional validation cohorts from OCAC and conducting additional experiments to evaluate its biological relevance with respect to treatment outcome, including the potential role of cholesterol metabolism as a determinant of response to chemotherapy. Our findings may provide some insight into the suggested role of statins in improving outcome for ovarian cancer.

